# Transcriptional Profiling of Resistant and Susceptible Cultivars of Grapevine (*Vitis L.*) Reveals Hypersensitive Responses to *Plasmopara viticola*

**DOI:** 10.3389/fmicb.2022.846504

**Published:** 2022-04-25

**Authors:** Peijie Gong, Jun Kang, Ehsan Sadeghnezhad, Ruoxuan Bao, Mengqing Ge, Yaxian Zhuge, Lingfei Shangguan, Jinggui Fang

**Affiliations:** Department of Horticulture, Nanjing Agricultural University, Nanjing, China

**Keywords:** downy mildew, grapevine, hypersensitive response, *Plasmopara viticola*, transcriptomic analysis

## Abstract

Grapevine downy mildew is the most serious disease of grapevine cultivars that affects the rate of resistance/susceptibility to *Plasmopara viticola*. In this study, we used the susceptible cultivar “Zitian Seedless” and the resistant cultivar “Kober 5BB” as materials to determine the transcriptome differences and phenotypes of the leaves after inoculation with downy mildew. The differences in microstructures and molecular levels were compared and analyzed. Fluorescence staining and microscopic observations confirmed that hypersensitive cell death occurred around the stomata in “Kober 5BB” infected by downy mildew zoospores. Meanwhile, transcriptomic profiling indicated that there were 11,713 and 6,997 gene expression differences between the resistant and susceptible cultivars at 72 h after inoculation when compared to control (0 h), respectively. The differentially expressed genes of the two cultivars are significantly enriched in different pathways, including response to plant-pathogen interaction, mitogen-activated protein kinase (MAPK) signaling pathway, plant hormone signal transduction, phenylpropanoid, and flavonoid biosynthesis. Furthermore, the results of functional enrichment analysis showed that H_2_O_2_ metabolism, cell death, reactive oxygen response, and carbohydrate metabolism are also involved in the defense response of “Kober 5BB,” wherein a total of 322 key genes have been identified. The protein interaction network showed that metacaspases (MCAs), vacuolar processing enzymes (VPEs), and Papain-like cysteine proteases (PLCPs) play an important role in the execution of hypersensitive responses (HR). In conclusion, we demonstrated that HR cell death is the key strategy in the process of grape defense against downy mildew, which may be mediated or activated by Caspase-like proteases.

## Introduction

The biotrophic oomycetes *Plasmopara viticola* is the causal agent of downy mildew disease in grapevine (*Vitis L*), which causes huge losses in quality, yield, and economic value (Wilcox et al., 2015; Li et al., 2016). *Plasmopara viticola* belongs to the *Peronosporales* Order, which is from *Oomycota* Phylum (Kamoun et al., 2015). It is one kind of plant-pathogen with strong specificity and is only parasitic in the living organs of grapevine (most cases in leaf and young fruit) (Gessler et al., 2011). The infection process of grape downy mildew usually occurs in a high-humid environment (above 85% humidity) with an optimum temperature of 22∼25°C (Gobbin, 2003). The degree of symptoms mainly varies with the surrounding environment and the resistance of the host grapevine (Atak et al., 2017; Frobel and Zyprian, 2019; Weitbrecht et al., 2021). Many studies demonstrated that different varieties of grapevine process different levels of resistance and susceptibility to downy mildew (Gomez-Zeledon et al., 2013; Zhang et al., 2017; Zhao et al., 2019; Heyman et al., 2021). Therefore, cultivating high-quality disease-resistant *Vitis* varieties is the best choice for sustainable viticulture. To improve this breeding strategy, it is necessary to understand in depth of the pathogenic mechanism of *P. viticola* when it invades the grape hosts.

To quarantine biotrophic pathogens at the site of infection, hypersensitive response (HR), as a special form of programmed cell death (PCD), could prevent the progression in plant hosts (Künstler et al., 2016; Balint-Kurti, 2019). In general, the plant’s innate immune system consists of two main immune processes (Jones and Dangl, 2006), containing the fundamental immune response called PAMP (Pathogen-Associated Molecular Patterns)-triggered Immunity (PTI) and a relative and highly evolved immune response called effector-triggered immunity (ETI). During the ETI process, the cells of the host plant performed rapid suicide through HR at the site of infection by the pathogen (Wang et al., 2018). HR could effectively inhibit the spread of pathogens and reduce the possibility of pathogen infection to other parts of the host (Heath, 2000), thereby improving the disease resistance of the host plant. However, in one plant species, the HR phenomenon does not exist in all genotypes or cultivars because it requires the long-term co-evolution of hosts and pathogens (Pontier et al., 1998). Therefore, it is often only manifested in a few resistant genotypes or cultivars. For instance, *Nicotiana* plants, containing the *N* resistance gene, formed the synchronous lesions more effectively than the wild type of tobacco in response to tobacco mosaic virus (TMV), and hypersensitivity improved the plant’s defense (Hatsugai et al., 2004; Wang et al., 2012). Moreover, HR-like phenotypes, as one kind of PCD, have also been described to enhance plant immunity in some other plant species, including tea plant, tomato, ginkgo tree, Arabidopsis, and grapevine (Greenberg and Yao, 2004; van Doorn and Woltering, 2005; Bellin et al., 2009; Wang et al., 2018; Li et al., 2019).

Regulatory mechanisms underlying HR can be different among various plant species. Compared to animals, higher plants do not own any proteins orthologous to caspases (Piszczek and Gutman, 2007; Opdenbosch and Lamkanfi, 2019), which is regulated and executed PCD in most fauna cases. Interestingly, caspase-like enzymatic activity has been found in plant PCD. With recent decades of identification work expanding, a type of cysteine-dependent proteases has finally emerged as the best candidate to replace caspases in plants (Lam and del Pozo, 2000). As of now, the identified proteases participating in PCD can be further classified into 3 protein families in plants: the legumain family-vacuolar processing enzymes (*VPEs*), the metacaspase family (*MCAs*), and Papain-like cysteine proteases (*PLCPs*) (Chichkova et al., 2004; Salguero-Linares and Coll, 2019).

Based on transcriptome data, the use of the bioinformatics approach is an effective method to study the mechanism of plant disease resistance (Naidoo et al., 2018). RNA-seq is a method based on next-generation sequencing technology to fully sequence the transcriptome (Simsek et al., 2017). Although the research on plant HR has made a lot of progress in the past ten years, the transcriptome analysis of grape susceptibility/resistance to downy mildew based on the HR phenomenon need to be considered in detail. Therefore, the investigation of biological processes regarding the network of up-streaming R genes, resistance-related genes, mitogen-activated protein kinase (MAPK) signaling pathway, and reactive oxygen species (ROS) with down-steaming caspase-like proteases can candidate critical points of HR in plants at transcriptional levels.

Here, we performed the transcriptome profiling at the early stages of interaction between the host and pathogen regarding the *Vitis* rootstocks “Kober 5BB” cultivar, which is resistant to *P. viticola* with HR phenomenon, and the table grape *V. vinifera* cv. “Zitian Seedless,” which is highly susceptible to the downy mildew. The key pathways and genes involved in apoptosis-related to two grapevine cultivars were discovered in three levels of signaling including perception, transduction, and response to *P. viticola* in a time-course experiment. According to our model, the results indicate the responsive genes and signaling pathways during downy mildew interaction, supplying new insights into the resistance mechanisms of grapevine to *P. viticola*.

## Materials and Methods

### Plant Material, Oomycetes Isolate, and Treatment

Three-year potted seedlings (resistant cultivar *Vitis* “Kober 5BB” rootstocks and susceptible cultivar *Viti’s vinifera cv.* “Zitian Seedless”) were used as host materials. The test plants were cultivated in the Baima grape test site of Nanjing Agricultural University (N30°10′, E120°5′) from September 2020. Before the experiment, to prevent the host plant’s self-carrying pathogen from interfering with the test results, the potted seedlings were lightly trimmed and then re-cultured in the artificial climate chamber.

The pathogen strain BS-4-MW was collected from the downy mildew localized in the Botanical Institute of Hohenheim University according to the described methods in previous reports (Thines et al., 2006; Kang et al., 2021). Mature sporangia of BS-4-MW was collected into a 1.5 ml Eppendorf tube from well-infected leaves and stored in a −80 freezer as inoculum. The sporangial suspension was prepared from one collected tube by adding sterile distilled water and the concentration was adjusted to 4 × 10^4^ sporangia/ml by using a hemocytometer (model 40449001, Fuchs-Rosenthal, Thomas, Germany) under a light microscope. Afterward, well-washed leaves were infected with sporangial suspension through their abaxial side and kept in a photo chamber (Model: PGX-460C-36L, Saifu, China) with high humidity at 21°C in darkness for 24 h. Then, the leaves were turned, placed on wet tissue paper with their abaxial side up, and further incubated under a photoperiod of 14 h light (25 μmol⋅m-2⋅s-1) with full-spectrum lamps and 10 h darkness (0 μmol⋅m-2⋅s-1) at 21°C. After about 7–10 days, the entire leaf was covered with freshly downy mildew. The inoculation method and conditions of the leaves were as described above, the third leaves of “Kober 5BB” and “Zitian Seedless” were sampled and pictures were taken by the camera (DFC400, Leica, Switzerland) at 1, 7, and 14 days post-inoculation (dpi). The non-wound inoculation of plants *in vivo* was used for RNA-Seq analysis as described in Jayaswall’s report (Jayaswall et al., 2016). Leaf tissues (third-fourth leaves) of “Kober 5BB” and “Zitian Seedless” were sampled at 0 h (with water treated to mimic the inoculated plants), 24 h, and 72 h post-inoculation (HPI), frozen in liquid nitrogen, and stored at −80°C. Three biological replicates were performed.

### Extraction of Total RNA, cDNA Library Construction, and Illumina Sequencing

A total of 18 total RNA samples were isolated using the cetyltrimethylammonium bromide (CTAB) method. The genomic DNA was digested using DNase (TAKARA, Dalian, China). The RNA concentration was measured using a NanoDrop 2000 spectrophotometer (Thermo Scientific, Wilmington, DE, United States). The purified messenger RNA (mRNA) and library construction were performed using an RNA library preparation kit (New England BioLbs, Ipswich, MA, United States) (NEB). The constructed library quality was determined using an Agilent 2100 bioanalyzer and an ABI StepOnePlus Real-Time PCR system, and the qualified cDNA library was sequenced on an Illumina Hiseq 2000 platform conducted by Novogene (Beijing, China^1^).

### Mapping of Reads and Analysis of Differential Gene Expression

All related information was transformed into raw data after online sequencing, removing the low quality, linker contamination, and reads with unknown base N content to obtain clean reads. The subsequently conducted analyses were all based on clean data with high quality. Clean reads were mapped to the *V. vinifera* reference genome^2^ using HISAT2^3^. Using the FPKM index (fragments per kilobase of transcript per million mapped reads) from remote scanning electron microscopy (RSEM) tools, gene and transcript expressions level were calculated and analyzed (Li and Dewey, 2011). Differential expression genes (DEGs) were determined based on the count values of each transcript between libraries using DESeq software (Anders and Huber, 2012). A *P*-value < 0.05 and | log2 (foldchange) | > 1 were set as thresholds for significant differential expression. Genes with FPKM < 0.3 were not considered as expressed genes and were therefore excluded from at least one group (Shangguan et al., 2017). The RNA-Seq raw data from this article can be found at the NCBI repository under the following accession number: GSE182149.

### Functional Annotations of Total DEGs

The differential expression genes of resistant cultivar *Vitis* “Kober 5BB” rootstocks and susceptible cultivar *Vitis vinifera cv.* “Zitian Seedless” was analyzed using online website tools and constructed a Venn diagram^4^. Gene ontology (GO) and Kyoto encyclopedia of genes and genomes (KEGG) pathway annotations were performed *via* cluster profile R package (Yu et al., 2012), with thresholds of FDR < 0.05 and *P*-value < 0.05, respectively. Functional annotation of all DEGs was also performed using MapMan *(Vvnifera 145^5^)* (Thimm et al., 2004). Heatmaps of the shared gene expression levels in both the resistant and susceptible grape plant cultivars were obtained using TBtools software (Chen et al., 2020).

### Construction and Analysis of Protein-Protein Interaction Network

The protein-protein interaction (PPI) network was constructed based on the data produced by the Search Tool for the STRING protein interaction database^6^ that search species was *Vitis vinifera*. The PPI network was visualized using the Cytoscape software (Smoot et al., 2011).

### Histochemical Staining and Microscope Observation of Infection Process of *Plasmopara viticola*

To determine and compare the phenotypic responses of the two varieties, photos of leaf disks were taken immediately after infection with pathogens or treatment with distilled water. We repeated the photography every 24 h until the fourth day; then, we continued the photography every 48 h until the tenth day, to follow up and observe the differences between the two cultivars during the infection process. In addition, the leaf disks were stained with Blankophor (Bayer, Leverkusen, Germany; 0.1% (w/v) 10% ethanol solution) at the same time (Kiefer et al., 2002) and the structure of the pathogen showed the specific fluorescence under the microscope. According to the specific operation method, first, we cleaned the observing leaf disk with distilled water and dried it with tissue paper (clean the non-infected side but avoid cleaning especially the inoculation part). In the next step, we placed the leaf disk with the inoculation side up and placed it on the microscope with a glass slide. For fluorescence staining observation, we treated the infected area with 30 μl Blankophor solution and used a fluorescence microscope (DM750, Leica, Switzerland) with a camera (DFC400, Leica, Switzerland) under ultraviolet light adjusted with DAPI mode (filtered light) Plate A, excitation filter BP 340—380 nm, interference filter LP 425. For picture analysis, we used Leica Application Suite software to save and analyze the results.

### Gene Expression Validation by Quantitative Real-Time PCR

For qRT-PCR, the RNA was extracted from grapevine leaves treated with *P. viticola* using the Trizol (Invitrogen) method as described in the instructions. The genomic DNA was digested using DNase (TAKARA, Dalian, China). The cDNA was prepared using the Hifair II 1st Strand cDNA Synthesis SuperMix for qRT-PCR (YEASEN, Shanghai, China). Specific forward and reverse primers were designed using Primer premier software and the qRT-PCR amplification reactions were performed on an ABI 7500 Real-Time PCR System (Applied Biosystems, United States) using SYBR Green (Vazyme, Nanjing, China) with three replicates. The *EF-1*α gene was used as an internal control (Monteiro et al., 2013). The relative expression levels were calculated using the 2-ΔΔCT formula (Schmittgen and Livak, 2008). The Minimum Information for Publication of Quantitative Real-Time PCR Experiments (MIQE) guidelines was followed for performing the qRT-PCR experiments (Bustin et al., 2009).

## Results

### Pathogenicity of *P. viticola* in Grapevine Cultivars

**FIGURE 1:**
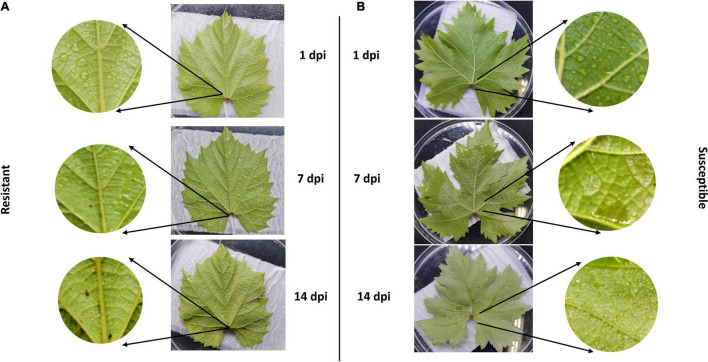
Relative-resistant (“Kober 5BB”) and relative-susceptible (“Zitian Seedless”) grape leaves symptom after downy mildew infection. Phenotypic characters and symptoms of grape downy mildew on leaves of resistant cultivar **(A)** *Vitis* rootstocks “Kober 5BB” and **(B)** *Vitis vinifera cv. “*Zitian Seedless” inoculated by *P. viticola* BS-4-MW.

We evaluated the rate of resistance among two cultivars including resistant and susceptible cultivars according to downy mildew symptoms. Our observation confirmed that inoculation of *P. viticola* sporangia suspension BS-4-MW strain on leaf belonged to the resistant cultivar *Vitis* “Kober 5BB” rootstocks was significantly less than the susceptible cultivar *Viti’s vinifera cv.* “Zitian Seedless” (Figures 1A,B). Pathogenicity tests showed that “Zitian Seedless” leaves had the typical downy mildew symptoms during pathogen infection (1, 7, and 14 dpi) (Figure 1B), but “Kober 5BB” leaves did not form visible sporangia during the inoculation process, instead of forming a few small necrotic spots (Figure 1A). This result confirmed the clear susceptible/resistance difference between two grape genotypes *via* the same *P. viticola* strain infection.

### Microscopic Observation of HR on Inoculated Leaves

**FIGURE 2:**
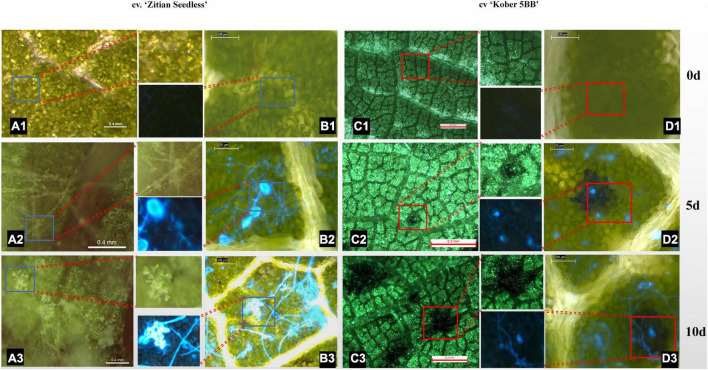
Leaf/Leaf disk symptoms of “Zitian Seedless” and “Kober 5BB” under different magnification of florence microscope after *P. viticola* BS-4-MW infection. **(A1–A3)** Symptoms of “Zitian Seedless” leaf abaxial side up were taken at 0, 5 and 10 d (days) after infection. **(B1–B3)** Sporangia on the same condition of **(A)** after blankophor staining. Blue frame is enlarged for revealing downy mildew sporangia on “Zitian Seedless”; **(C1–C3)** Symptoms of “Kober 5BB” leaf abaxial side up were taken at 0, 5 and 10 d (days) after infection. **(D1–D3)** Sporangia on the same condition of **(C)** after blankophor staining. Red frame is enlarged for indicating HR spots on “Kober 5BB”; The scale bar in the picture and the corresponding length unit reflects the objective size.

To confirm whether the HR spots are located in the infection area and involved in grape resistance to *P. viticola* BS-4-MW, the leaves of *Vitis* “Kober 5BB” rootstocks, and *V. vinifera cv.* “Zitian Seedless” were inoculated with sporangia suspensions and, then, stained with Blankophor standing solution. Based on the result of Figure 1, we chose 10 dpi to observe the difference in leaf disk symptoms between resistant and susceptible grapes *via* a Florence microscope. As shown in Figures 2A1–A3, the sporangia are shown on the surface of the “Zitian Seedless” leaf disk gradually since 5 dpi (days post-infection), but was not observed on “Kober 5BB” during the whole process. However, the formation of necrotic sites could be observed on the “Kober 5BB” disks around the stomata area since 5 dpi, the starting position for downy mildew to infect the host (Figures 2C1–C3). Also, the “Kober 5BB” leaves exhibited thickening of the cell walls and epidermal cell necrosis, and part mesophyll cells became necrotic as well (Figures 2D1–D3). Furthermore, under the DAPI field, to see the specific fluorescence of downy mildew, the results showed that “Zitian Seedless” mycelium and sporangia gradually grow and spread around the many areas of leaf disk (Figures 2B1–B3), whereas “Kober 5BB” did not show sporangia formation with very rare mycelium growth (Figures 2D1–D3). Besides, we took five-time points (dncysted zoospores, 1, 3, 5, and 7 dpi) to photograph the morphological structure of *P. viticola* in the two grape varieties, including zoospore germinated tube, invasive hyphae, haustoria, and sporangium (Supplementary Figure 1). We observed a complete infection process in “Zitian Seedless” with many sporangia formations observed at 7 dpi. In “Kober 5BB,” even with zoospore germination, invading hyphae, and successive appearance of haustoria (within 3 dpi), the distribution of mycelium was relatively limited since the death site appeared from 3dpi, and very few sporangia were observed at 7 dpi. Overall, these results suggest that hyphae successfully infected “Zitian Seedless” leaves *in vivo* but did not elicit an HR. Moreover, the HR is associated with “Kober 5BB” resistance to *P. viticola* BS-4-MW.

### *Plasmopara viticola* Uniquely Alters the Transcriptomic Profiling of Resistant and Susceptible Cultivars in Grape

**FIGURE 3:**
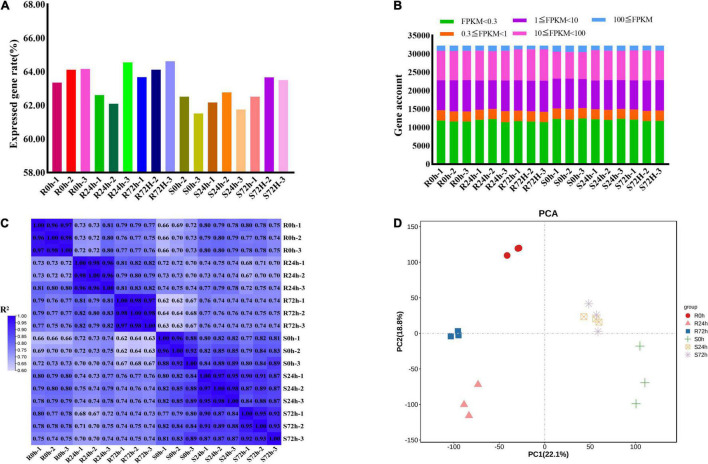
Different samples analysis and gene expression information. **(A)** Rate of expressed genes based on all genes. **(B)** Gene numbers of different fragments per kilobase of transcript per million mapped reads (FPKM) values. **(C)** Correlation matrix showed the correlation between samples. **(D)** Principal Component Analysis (PCA) were performed on the biological replicates of each sample. R resistant cultivar *Vitis* “Kober 5BB” rootstocks. S *Vitis vinifera cv*. “Zitian Seedless”.

In total, 826,124,582 raw data reads were generated from 18 samples, and the Q30 (%) of all samples (the percentage of bases with a base recognition accuracy rate above 99.9%) was higher than 94.05%. Then, the raw read sequences were filtered and passed through quality control. In total, 801,100,242 clean data containing 120.19 G nucleotide sequences were obtained with rates of more than 96.97%. Next, more than 87.03% of high-quality reads from individual sample types were mapped to the grapevine reference genome, of which 84.89% or more were mapped uniquely (Supplementary Table 1). Only 61.51 to 64.52% of genes (32,247 genes in total) showed an FPKM value ≥ 0.3 (Figures 3A,B and Supplementary Tables 2, 3). More than half of the genes had transcriptional values exceeding 1 (Figure 3B and Supplementary Tables 2, 3).

To check the quality of data and obtain an overview of variations among all samples, the degree of sample repeatability was evaluated according to the R^2^ value. Overall, all biological repetitions showed highly correlated expressions (Figure 3C). In addition, principal components analysis (PCA) was conducted using RPKM values. Principal component (PC) 1 explained 22.1% of the variation and PC2 comprised 18.8% of the variance, which combined analysis of variance revealed 40.9% among 6 groups (18 samples). Using transcriptomic data, the resistant (R) and susceptible (S) accessions were separately classified into two groups. Therefore, transcriptomic data regarding both R and S genotypes indicated the *P. viticola* BS-4-MW treated samples have considerable variations in comparison with mock in response to pathogen attack. Furthermore, PCA plots revealed very little to no variance among all sample replicates and confirmed the high reproducibility of biological replicates and high-quality sequencing data (Figure 3D).

### Validation of Differential Expression Data and Temporal and Spatial Expression Analysis in Resistant/Susceptible Cultivars

**FIGURE 4:**
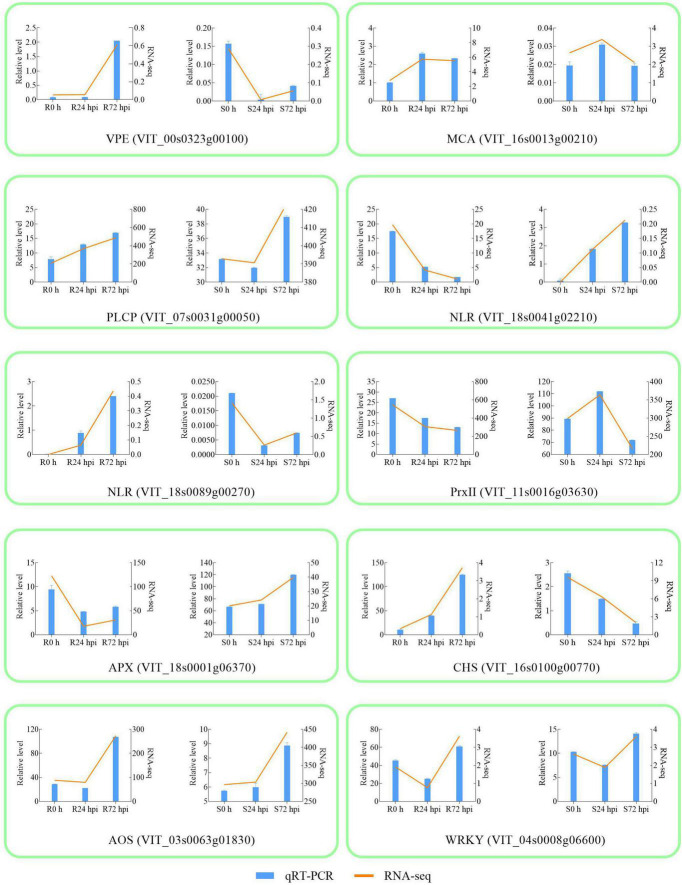
Validation of RNA-seq by qRT-PCR. The column chart and the main longitudinal coordinate represent the relative expression of quantitative real-time PCR (qRT-PCR), while the broken line diagram and the secondary longitudinal coordinate represent the FPKM value of RNA-seq. VPE Vacuole processing enzyme; MCA metacaspase; PLCP Papain-like cysteine protease; NLR Nucleotide-binding domain and Leucine-rich Repeat; PrxII type-2 peroxiredoxin; APX ascorbate peroxidase; CHS chalcone synthase; AOS allene oxidase synthase; R resistant cultivar *Vitis* “Kober 5BB” rootstocks. S *Vitis vinifera cv.* “Zitian Seedless”.

To validate the RNA-Seq results, 10 DEGs were randomly selected for qRT-PCR analysis. Gene-specific qRT-PCR primer pairs are listed in Supplementary Table 4. The expression results showed that the expression patterns were similar between the qRT-PCR and RNA-Seq data at different times post-inoculation, suggesting reliable expression data by RNA-Seq (Figure 4). The three HR and caspase-like genes (*VPE*, *MCA*, and *PLCP*) showed different trends in resistant and susceptible cultivars. Among the resistant cultivars, *VPE*, *MCA*, and *PLCP* were upregulated at 72 hpi compared to 0 h. At 72 dpi, *VPE* expression showed a decreasing trend, while the expression of *MCA* and *PLCP* showed an increasing trend in susceptible cultivars. Although *MCA* and *PLCP* showed an upregulated expression trend in susceptible cultivars, their expression levels were lower than those in resistant cultivars. In addition, allene oxidase synthases (AOS) and *WRKY* were upregulated in resistant and susceptible cultivars at 72 hpi, while chalcone synthase (CHS), NLR (VIT_18s0041g02210), NLR (VIT_18s0089g00270), and ascorbate peroxidase (APX) showed opposite expression trends, and type-2 peroxiredoxin (PrxII) was downregulated in both cultivars. The above results indicated that 10 genes responded to *P. viticola* in both resistant and susceptible cultivars, in particular HR and caspase-like genes (*VPE*, *MCA*, and *PLCP*) deserve further research.

### DEGs Behave Based on the Time Course in Both Resistance and Susceptible Cultivars

In our study, the significant DEGs were used to further analyze their function in both cultivars of grapevine in response to *P. viticola* BS-4-MW. A total of 11,713 and 6,997 DEGs were identified in the inoculated leaves of “Kober 5BB” and “Zitian Seedless,” respectively (Supplementary Figure 2). Compared with the unigene transcription at 0 h (control), 4,434 and 4,000 DEGs were up- and downregulated in “Kober 5BB” at 24 hpi, respectively; however, 2,357 and 2,278 DEGs were up- and downregulated in “Zitian Seedless,” respectively. The number of up- and downregulated DEGs reached 4,198 and 3,726 DEGs in “Kober 5BB” at 72 hpi respectively; however, 2,346 upregulated DEGs and 2,421 downregulated DEGs were identified in “Zitian Seedless” (Supplementary Figure 2).

**FIGURE 5:**
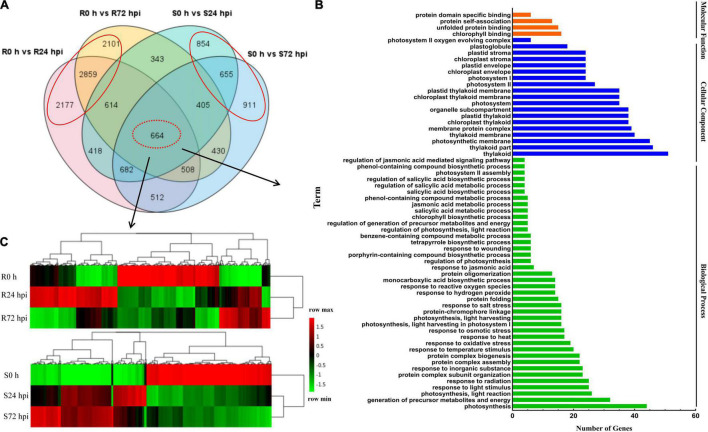
Shared genes in both of resistant and susceptible grape leaves. **(A)** Venn diagram of DEGs in “Kober 5BB” and “Zitian Seedless” leaves at 24 and 72 hpi, respectively. **(B)** GO functional classification of the shared genes in both “Kober 5BB” and “Zitian Seedless” leaves. **(C)** Clustering analysis of the shared genes in both the “Kober 5BB” and “Zitian Seedless” leaves. Each row represents an inoculation time point. Each column represents a gene. Expression values with up-regulation to down-regulation vary from red to green. R resistant cultivar *Vitis* “Kober 5BB” rootstocks. S *Vitis vinifera cv*. “Zitian Seedless”.

To identify the resistance mechanisms in response to downy mildew, all the DEGs were assessed in both the resistant and susceptible grape cultivars at 24 and 72 hpi. According to the Venn diagram, the 664 DEGs are involved in both “Kober 5BB” and “Zitian Seedless” leaves in the response to *P. viticola* BS-4-MW during the infection progress (Figure 5A). Using GO analysis, these DEGs were significantly enriched in 63 terms including; 40 biological processes, 19 cellular components, and 4 molecular functions; which the DEGs were mainly enriched in the functional pathway that was mostly associated with disease resistance, such as the response to phytohormones (jasmonic acid and salicylic acid), reactive oxygen species, transmembrane transporter activity, biotic stimulus, and signaling (Figure 5B and Supplementary Table 5).

Heat map and cluster analysis of 664 shared DEGs displayed three main groups according to their expression patterns in both the resistant (R0 h, R24 hpi, and R72 hpi) and susceptible (S0 h, S24 hpi, and S72 hpi) grape cultivars (Figure 5C). For the upregulated DEGs in the resistant cultivar “Kober 5BB,” R24 hpi had the most genes (310), followed by R72 hpi (142). Meanwhile, most genes in the susceptible cultivar “Zitian Seedless” were classified into S72 hpi (252), followed by S24 hpi (210). For the downregulated DEGs in the “Kober 5BB,” R72 hpi had the most genes (326), followed by R24 hpi (256). In the “Zitian Seedless,” most genes were enriched in S24 hpi (310), however, the S72 hpi was least enriched in the number of genes (292). Besides, at 0 h, there were some genes with relatively high basal expression in R and S, namely R 0 h (282) and S0 h (345). Overall, the different types of defense systems were involved in both the resistant and susceptible grape plant cultivars in the response to downy mildew.

### Function of DEGs Conduct Resistance Mechanisms More in Resistance Cultivar After *P. viticola* Infection

**FIGURE 6:**
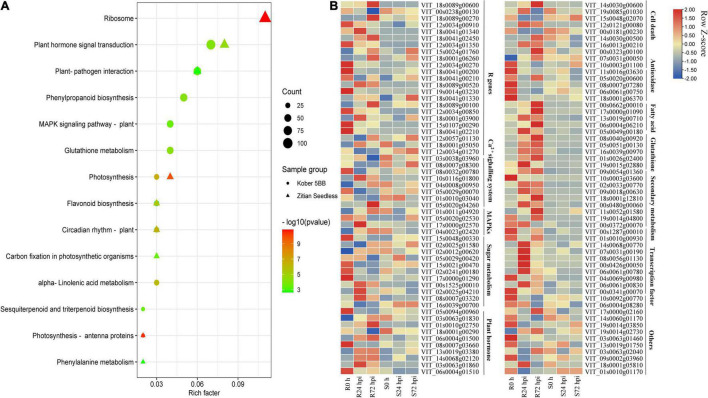
DEGs specific to resistant grape inoculated with *P. viticola* BS-4-MW. **(A)** KEGG significantly enriched classifications of the 7137 and 2420 DEGs specific to resistant and susceptible grape inoculated with *P. viticola* BS-4-MW.; **(B)** heatmap of some key differential expression genes (DEGs) expressed specifically in the resistant grape cultivar. R resistant cultivar *Vitis* “Kober 5BB” rootstocks. S *Vitis vinifera cv*. “Zitian Seedless”.

To identify the function of identified DEGs in Figure 5A, the KEGG analysis was performed. In the susceptible grape cultivar, 2,420 DEGs were enriched in 116 pathways and 7,137 DEGs in the resistant cultivar were enriched in 117 pathways. Among determined pathways, plant-pathogen interaction, MAPK signaling pathway, plant hormone signal transduction, glutathione metabolism, sesquiterpenoid, and triterpenoid biosynthesis, alpha-Linolenic acid metabolism, flavonoid biosynthesis, and Phenylpropanoid biosynthesis were markedly enriched (*P* < 0.01; Figure 6A), suggesting that these eight pathways play important roles in grape plant’s defense against downy mildew. Among the 7,137 DEGs, 322 genes associated with disease resistance including pathogen receptors (23); antioxidase (13); signaling transduction such as Ca^2+^ signaling (8) and MAPK signaling systems (4); sugar metabolism (11); secondary metabolites (60); fatty acids (20); glutathione (26); transcription factors (TFs) (119); the cell death (15) and plant hormone (10), as well as other defense function genes (13), were identified. The expression of all key genes was significantly changed in “Kober 5BB,” but slight change or opposite expression patterns was observed in “Zitian Seedless” during *P. viticola* BS-4-MW infection (Figure 6B). Information on the 322 genes associated with disease resistance was listed in Supplementary Table 6.

### Enrichment Analysis Highlights the Pathways Related to Plant Disease Resistance in Resistant Cultivars, Not Susceptible

**FIGURE 7:**
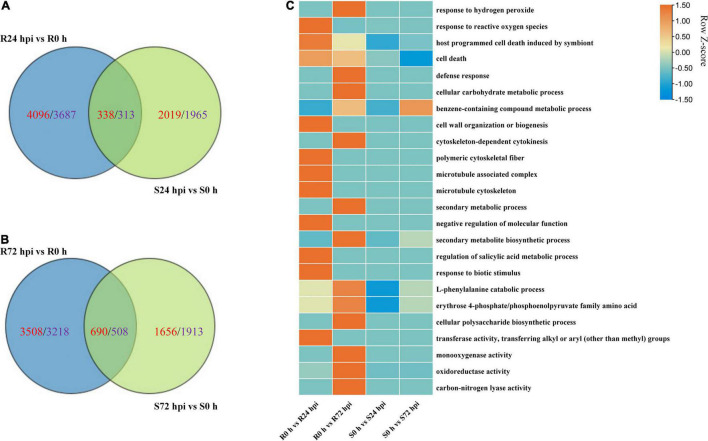
Change in DEGs in resistant “Kober 5BB” and susceptible “Zitian Seedless” grape cultivars inoculated with *P. viticola* BS-4-MW during the infection process in comparison with their respective controls (0 h). **(A,B)** Venn diagram of the DEGs in “Kober 5BB” and “Zitian Seedless” leaves at 24 and 72 hpi, respectively; **(C)** Enriched GO terms related to disease resistance identified from DEGs. Terms are considered enriched when *P* < 0.05. R resistant cultivar *Vitis* “Kober 5BB” rootstocks. S *Vitis vinifera cv*. “Zitian Seedless”.

The Venn diagram of the up- and downregulated DEG data of both cultivars shows that 4,096 and 2,019 upregulated DEGs, as well as 3,687 and 1,965 downregulated DEGs, were specifically expressed in “Kober 5BB” and “Zitian Seedless” at 24 hpi, respectively (Figure 7A). More specific upregulated DEGs were identified in “Kober 5BB” (3,508 DEGs) than “Zitian Seedless” (1,656 DEGs) at 72 hpi, while the number of specific downregulated DEGs decreased and increased in the “Kober 5BB” (3218) and the “Zitian Seedless” (1913), respectively (Figure 7B). The GO analysis indicated that these DEGs were enriched in multiple pathways associated with disease resistance. The number of DEGs changed during the infection process (*P* < 0.05; Supplementary Figure 3). In particular, DEGs were enriched in ROS (response to hydrogen peroxide, benzene-containing compound metabolic process, defense response, cellular carbohydrate metabolic process, and response to reactive oxygen species), cytoskeleton (cell wall organization, cytoskeleton-dependent cytokinesis, polymeric cytoskeletal fiber, microtubule-associated complex, and microtubule cytoskeleton), secondary metabolic process, regulation of biological process (secondary metabolite biosynthetic process, negative regulation of molecular function, regulation of salicylic acid metabolic process, response to biotic stimulus, and L-phenylalanine catabolic process), and protease activity (erythrose 4-phosphate/phosphoenolpyruvate family amino acid, mono-oxygenase activity, oxidoreductase activity, acting on paired donors, with incorporation or reduction of molecular oxygen, transferase activity and carbon-nitrogen lyase activity) in “Kober 5BB,” and the number of DEGs significantly increased during *P. viticola* BS-4-MW infection. In contrast, these DEGs were not enriched in the susceptible cultivar, and the number of DEGs decreased (Figure 7C). Taken together, the results revealed that PCD and ROS play important roles in grape plant defense against downy mildew.

### MAPK Signaling Pathway Acts in Resistance Cultivar in Response to *P. viticola*

**FIGURE 8:**
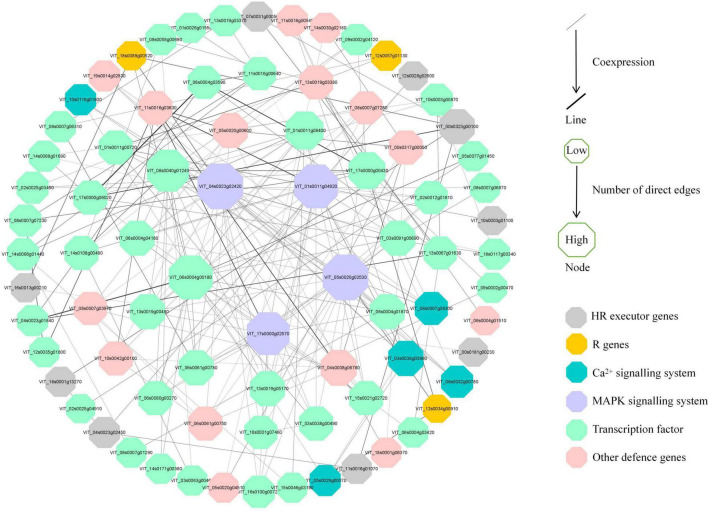
Interaction analysis of putative DEGs that are associated with disease resistance and are involved in grape defense to *P. viticola* BS-4-MW.

To further investigate the regulatory pathways in response to *P. viticola* BS-4-MW, a PPI network was constructed according to the significant DEGs in “Kober 5BB” (Figure 8). The network analysis was performed and the average number of direct neighbors in the network for each gene was 4.974. The figure consists of four layers of concentric rings. And the first outer layer of rings represents the interaction with the other three layers. The closer the proteins appear in the diagram to the center (Figure 8), the more important their roles are in the overall reaction pathway. In this network, MAPKs; Ca^2+^ signaling; TFs; pathogen receptors (resistance genes, R genes); and HR executor genes (hypersensitive response), such as Papain-like cysteine proteases (*PLCPs*), metacaspase-like regulator (*MCP1*), and vacuolar processing enzymes (*VPEs*), were significantly correlated. Four hub nodes (MAPK) were involved in grape plant defense against *P. viticola* BS-4-MW, which suggested the MAPK signaling pathway is the crucial signal transduction pathway. Furthermore, HR executor genes were the major PCD regulator of *P. viticola* BS-4-MW infection, as 9 nodes interacted with major signaling molecules and other defense genes (Figure 8).

## Discussion

**FIGURE 9:**
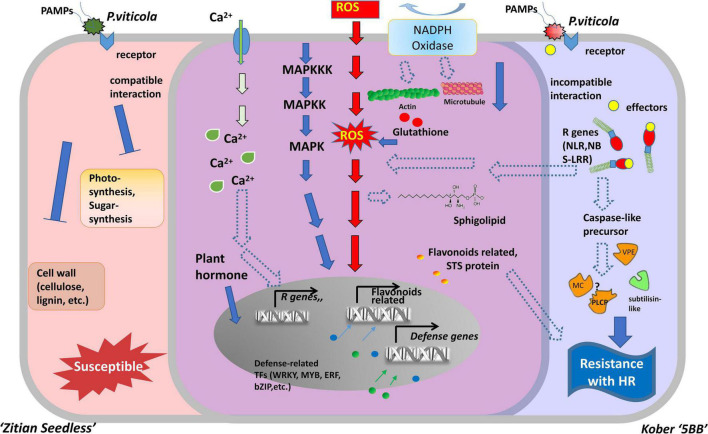
Model for two types of cells belonged to susceptible and resistance grape in response to downy mildew strains BS-4-MW. The diagram represents some of the characteristic features of two different grape cells during *P. viticola* infection. Left side (red) is as susceptible cell of *Vitis vinifera cv.* “Zitian Seedless” grape. Right side (blue) is as resistant cell of *Vitis* “Kober 5BB” rootstocks grape accomplices with HR-like cell death. The middle overlapping part (purple) is some signal pathway shared both in the susceptible/resistant cells. R gens Resistant genes; NBS-LRR nucleotide-binding site, leucine-rich repeats, as R receptors to recognize effecters; MAPK Mitogen- activated protein kinase; NADPH nicotinamide adenine dinucleotide phosphate hydrogen; Ca^2+^, Calcium ion; STS, Stilbene synthase; VPE, Vacuole processing enzyme; MC, Metacaspase; PLCP, Papain-like cysteine protease; ROS, reactive oxygen species; TFs, Transcription factors; HR, hypersensitive response.

Necrotrophic pathogens infect and kill host tissue and extract nutrients from the dead host cells directly, while most hemibiotrophic and entire biotrophic pathogens colonize living plant tissue and obtain nutrients sustainably (Freeman and Beattie, 2008; Fawke et al., 2015). Therefore, in many cases, the ETI in the host plant culminates in the HR, providing an efficient strategy to block pathogens (Heath, 2000; Jones and Dangl, 2006). Based on a long co-evolution history of host plants and pathogens, HR is always associated with a high degree of plant resistance (Salguero-Linares and Coll, 2019). In the interaction between grape and downy mildew, the relevant mechanism of cell death caused by HR, especially at the transcriptional level, is still unclear. In this study, we used RNA-seq to analyze the DEGs in a resistant cultivar and compare them with a susceptible cultivar during the time course after infection with *P. viticola.* Our results indicated that the improvement of disease resistance needs the activation of genes involved in the different levels of molecular, metabolic, and physiological responses. The experimental results revealed that the HR and caspase-like genes (*VPEs, MCAs*, and *PLCPs*) play crucial roles in improving disease resistance. These defense responses might be mediated by multiple transcriptional factors, R genes, Ca2 + signaling, and the MAPK signaling pathway. Besides, secondary metabolites biosynthesis pathway, especially flavonoid biosynthesis are also involved in grape HR formation after *P. viticola* infection (Figure 9).

### Necrotic Spots Were Localized in the Guard Cells of Stoma in Resistance Cultivar

In our laboratory, downy mildew strains were preserved *in vivo* and used as the pathogen test materials for the *V. riparia* grape hybrid progeny “Kober 5BB” as a resistant cultivar. After 7 dpi, it began to be accompanied by obvious HR necrotic spots on the leaves (Figure 1). According to our repeated experiments, this result is much later than the previously reported results (Casagrande et al., 2011; Liu et al., 2015; Gong et al., 2019), which showed HR necrosis can be seen with naked eyes about 3—5 days after pathogen inoculation. We infer that this is mainly due to the different strains of downy mildew. Since HR is a rapid and localized plant cell death induced by rust fungi, it becomes visible as necrotic spots at the site of infection with brown lesions belonging to dead cells (Heath, 2000). The scarification of infested cells by PCD protects the other cells and healthy tissue against pathogen progression. This means that in non-compatible interactions between host and pathogen, necrotic spots appear on the part of the host infected by the pathogen (Hatsugai et al., 2004; Wang et al., 2012). In this study, we observed and recorded the morphology of inoculated leaves in the susceptible/resistant grapes after inoculation. The results indicated that the HR spots of downy mildew *P. viticola* on “Kober 5BB” were located on the guard cells of the stoma and some of the mesophyll cells around it within 96 h after inoculation. Using the DAPI staining method, the formation of sporangia was almost not found on the “Kober 5BB” and only a small amount of hyphae was observed. In addition, we observed Zoospore germination, invasive hypha, and especially haustoria formation when occurred before HR dead sites appeared in the infected “Kober 5BB” (Supplementary Figure 1). Visible haustoria are the key to the host specificity by delivering the AVR effectors that will be recognized by the specific R genes (NLRs) in resistant plants to induce HR responses (Diez-Navajas et al., 2008). Therefore, HR death occurred specifically in the stomata infested by downy mildew zoospores and suppressed the zoospores germination in the stomata. At the same time, related executive genes involved in the HR phenomenon such as *PLCPs*, *VPEs*, and *MCAs* have also been identified (Supplementary Table 6). In our previous work, the *VPEs*, *PLCPs*, and *MCAs* genes have been preliminarily identified in the interaction between grapes and pathogens (Salguero-Linares and Coll, 2019). Among them, *VPEs* can promote the hydrolysis of the protein in the vacuole, which leads to the rupture of the vacuole and initiates the PCD of plant cells (Hatsugai et al., 2015). *PLCPs* are responsible for the degradation of intracellular proteins and regulate PCD (Kang et al., 2021). *MC2* and *MC5* genes mediate ETI-like cell death in grapevine defense against infection of *P. viticola* (Gong et al., 2019). These results indicate that these genes play an important role in regulating the HR phenomenon.

### Innate Immunity Requires NBS-LRR Receptors to Restrict Downy Mildew Infection

At the initial stage of infection, downy mildew pathogen secretes various virulence factors into host cells to facilitate successful invasion. Plant R genes are involved in different networks of plant-pathogen interactions, as they enable plants to recognize pathogens and activate inducible defenses (Kourelis and van der Hoorn, 2018). The vast majority of plant R genes are NBS-LRR, as they encode proteins with an amino-terminal variable domain, a central Nucleotide Binding Site (NBS), and a carboxy-terminal Leucine-Rich Repeats (LRR) domain (Meyers et al., 1999; Wang et al., 2020). In our study, 23 R genes (22 NBS-LRRs and 1 PRR), specific to the resistant grape plant, were identified, and the expression of these genes was significantly induced by *P. viticola* (Supplementary Table 6 and Figure 6B). Goyal et al. (2020) reported that 63 NBS-LRRs are also involved in grape plant defense against powdery mildew. These findings suggest that multiple R genes are involved in grape plant’s defense. In addition, NBS-LRR receptors can activate the immune response of plants (ETI) by recognizing specific pathogenic effector proteins (non-toxic factors), producing an allergic reaction (HR), and locally killing pathogens in infected cells (Cui et al., 2015). Interestingly, we observed that these 11 R genes were upregulated in the resistant grape plant and 7 R genes were upregulated in the susceptible cultivar. Therefore, we hypothesized that NBS-LRR receptors may be the upstream factors of the signaling pathway of HR in “Kober 5BB,” as a resistant variety, and activate the innate immunity to restrict downy mildew infection (Figure 9).

### Effector-Triggered MAPK and Ca^2+^ Signaling Pathways as a Central Hub Are Necessary for Downy Mildew Resistance

Plant MAPK cascades play pivotal roles in signaling plant defense against pathogen attack (Meng and Zhang, 2013). MAPK cascades typically consist of three protein kinases, MAPK, MAPK kinase (MAPKK), and MAPK kinase (MAPKKK) (Jonak et al., 2002; Cakir and Kilickaya, 2015). ETI frequently results in HR cell death, and this process is inseparable from the MAPK cascades and Ca^2+^ signaling pathways. They are activated by HR executor genes and the R gene (Devendrakumar et al., 2018). In this study, we obtained four MAPK genes, some were significantly induced by *P. viticola* BS-4-MW, some were highly expressed before infection, and directly interacted with HR and R genes using the PPI network. Meanwhile, eight Ca^2+^ signaling-related genes were identified and five of these genes interacted with HR genes and other defense-related genes (Supplementary Table 6 and Figures 6B, 8). These results demonstrated that the MAPK and Ca^2+^ signaling may be mediated by HR and R genes following downy mildew pathogen infection, and MAPK cascades constitute the main defense signaling pathway (Figure 9). In addition, the expression of MAPK7 and MAPK9 was significantly upregulated in the resistant grape plant cultivar following *P. viticola* BS-4-MW infection, but was downregulated in the susceptible cultivar (Figure 6B). Interestingly, the study by Chakraborty et al. (2019) indicated that physical interaction and phosphorylation by MAPK9 protect the degradation of WRKY40 that induces resistance response in chickpea to *Fusarium* wilt disease by modulating the transcription of defense responsive genes. Overexpression of Arabidopsis MAPK7 led to activation of plant basal and systemic acquired resistance was confirmed in the study of Zhang et al. (2007). These results suggest that the specific upregulated expression of MAPK7 and MAPK9 may play an important role in grape plant defense against downy mildew pathogen.

### Reactive Oxygen Species’ Burst and Scavenging Amplify the Resistance to Disease

Reactive oxygen species are an effective weapon that can be produced rapidly and utilized against pathogen infection. The production of ROS in plant cells is a hallmark of successful recognition of plant pathogens and activation of plant defenses (Levine et al., 1994; Lamb and Dixon, 1997; Torres, 2010). As a signaling molecule, ROS can regulate PCD in plants during pathogen infection and can mutually regulate MAPK signaling (Gechev et al., 2006; Daudi et al., 2012; Wang et al., 2018). Rahman et al. (2019) reported that the HR and ROS bursts are involved in grape plant defense against *Botrytis cinerea*. Pathogen-induced ROS generation in chloroplasts was known to play a crucial role in the execution of HR cell death in plants (Liu et al., 2007). In our study, we observed that DEGs were enriched in the H_2_O_2_ catabolic process (Figure 7C). H_2_O_2_ production is one of the earliest (12 hpi) detectable cytological events against downy mildew in the resistant grapevine cultivar “Solaris” (Nogueira Junior et al., 2020). These results showed that H_2_O_2_ may play an important role in grape plant defense against multiple diseases, including downy mildew pathogen, and H_2_O_2_ production may be regulated by MAPK signaling (Figure 9). Previous studies demonstrated that pathogen infects the host and leads to an oxidative burst mediated by NADPH oxidases (Rboh) (Cazale et al., 1998; Park et al., 1998). Changes in the expression of NADPH oxidases provoked through perturbed ROS homeostasis suggest that transcriptional activation of certain NADPH oxidases is an essential intermediate step in the activation or amplification of defense responses (Mittler et al., 2004). We discovered that Rboh, which is induced by downy mildew pathogen, is enriched in H_2_O_2_ catabolic processes and that the expression of this gene is distinctly upregulated both in the resistant grape plant cultivar and in the susceptible cultivar (Figure 6B). Interestingly, Torres et al. (2002) research confirmed the absence of the NADPH oxidase genes, AtRbohD and AtRbohF, suppress ROS production and the defense response of Arabidopsis against pathogen attack. ROS levels depend on the balance between ROS production and scavenging, and excess ROS could injure cells (Mittler et al., 2004; Liu et al., 2021). ROS amounts depend both on enzymatic and non-enzymatic scavenging molecules such as PrxII, APX, dehydroascorbate reductase (DHAR), and 1-Cys peroxiredoxin (1-CysPrx), which offer a highly efficient system for maintaining ROS homeostasis (Supplementary Table 6). Peroxiredoxins (Prxs) have emerged as important factors linking reactive oxygen species (ROS) metabolism to redox-dependent signaling events (Camejo et al., 2015). In our analysis, the higher expression of three Prxs (two PrxIIs and one 1-CysPrx) in “Kober 5BB,” relative to “Zitian Seedless,” may contribute to resistance in “Kober 5BB.” APX is a key enzyme in the ascorbate-glutathione cycle, an important antioxidant system that can detoxify ROS in plant cells (Liu et al., 2021). In grapevine, APX is induced by pathogen infection (Marsh et al., 2010). Notably, we observed that APX has higher levels of basal expression than the Susceptible cultivar, which probably contributes to a pre-formed resistance of this resistant cultivar, it is already in a state of defense or resistance before the pathogen arrived and may also be associated with Redox homeostasis. Feng et al. (2014) confirmed that the DHAR knockdown resulted in a much greater H_2_O_2_ accumulation, together with wheat’s higher infections to stripe rust (Feng et al., 2014). We found that DHAR was higher in basal expression (0 h) in the resistant grape plant cultivar, then, rapidly peaked and decreased. These results imply that DHAR may play a role in preventing the accumulation of H_2_O_2_ in the early stage of downy mildew infection.

### Carbohydrate Metabolism and Phenylpropanoids Pathways Are Requirements for the Grape Downy Mildew Resistance

In plant-pathogen interactions, carbohydrate metabolism plays several roles, including energy source for the activation of defense reactions, regulators of resistance-related genes expression (sugar signaling), and nutrients (Roitsch et al., 2003; Bolton, 2009; Liu et al., 2021). They can induce pathogenesis-related proteins and sink specific genes (such as invertase) (Bolouri Moghaddam and Van den Ende, 2012). More DEGs were enriched in carbohydrate metabolism in the resistant grape plant cultivar than susceptible grape plant cultivar following *P. viticola* infection (Figures 5B, 7C), and the expression of some specific genes associated with carbohydrate metabolism was significantly upregulated (Figure 6B). Lecompte et al. (2017) research confirmed that the adjustment of glucose metabolism plays a determinative role in plant defense during necrotrophic interaction of *B. cinerea* and *Sclerotinia sclerotiorum* with tomato. In our study, five enzymes related to glucose metabolism were specifically induced in “Kober 5BB.” The specific expression of these enzymes enhances the activation of glycolytic pathways, which lead to the production of ATP and NADPH in response to pathogen infection (Liu et al., 2021). At the same time, the expression levels of key genes associated with flavonoids and the phenolics biosynthesis pathway, including the CHS and proteolytic phenylalanine ammonia-lyase regulator (KFB-PAL), were upregulated in resistant cultivars. In the present study, DEGs involved in phenylpropanoid and flavonoids metabolism were enriched in the resistant grape plant, and the expression levels of CHS and KFB-PAL increased by *P. viticola* (Figure 6B). These results were similar to those of Fofana et al. (2002) and Oliva et al. (2020), in which the increase of phenylalanine levels in leaves reduced susceptibility to *Botrytis cinerea* and the resistance of cucumber plants to powdery mildew is related to the accumulation of chalcone synthase. In general, sugars as sources of carbon are essential to fuel the energy required for defenses and serve as signals for the regulation of defense genes in plant-pathogen interactions. Pathogens and lipid metabolites in the host play a key role in the expression of plant pathogenesis and defense responses (Shah, 2005). Fatty acids are substrates for the biosynthesis of oxidized lipids. In addition, fatty acids regulate the activity of enzymes involved in the generation of signal molecules in plant defense (Weber et al., 1997; Wang and Wang, 2001). Sequeira (1983) research confirmed that pretreatment with fatty acids confers a faster and stronger induction of expression of defense-associated genes and reduces the severity of the HR in pepper plants inoculated with *Xanthomonas campestris*. Our results showed that most genes associated with fatty acid metabolism were detected, and the expression of these genes was higher in the resistant grape plant than in the susceptible grape plant cultivar. In contrast, these genes were either downregulated or not regulated in the susceptible cultivar (Figure 6B). Interestingly, in Arabidopsis, infection by *P. syringae* caused the accumulation of fatty acids, which the application of fatty acids to Arabidopsis leaves led to cell death and induced expression of the glutathione-S-transferase (Vollenweider et al., 2000). In this study, we found some glutathione S-transferase that is specifically expressed in “Kober 5BB” (Figures 6B, 9). Although the previous report confirmed that fatty acids influence pathogenesis and resistance mechanisms associated with plant-pathogen interactions (Christensen and Kolomiets, 2011), the role of fatty acids in the interaction between grape plant and downy mildew needs to be further explained.

### Stress Hormones and Regulatory Factors Associated With Grape Resistance in Response to Downy Mildew

Plant hormones have pivotal roles in the regulation of plant growth, development, and reproduction. Furthermore, they emerged as cellular signal molecules with key functions in the regulation of immune responses to pathogens (Robert-Seilaniantz et al., 2011; Pieterse et al., 2012). Among different hormones, salicylic acid (SA) and jasmonic acid (JA) as mediators of signaling pathways form the backbone of the plant defense system (Sherif et al., 2016; Gupta et al., 2020). In our study, the shared genes in the two cultivars were enriched in salicylic acid and jasmonic acid-related pathways (Figure 5B). Meanwhile, six genes involved in the resistant grape plant including receptor complex-component COI, jasmonoyl-amino acid hydroxylase, two AOS, and two 13-lipoxygenases, were induced by *P. viticola* (Supplementary Table 6), suggesting that these genes act in the regulation of plant hormones and defense responses. Transcription factors (TFs) can regulate the expression of multiple genes related to pathogens and improve the disease resistance of plants (Gordan et al., 2011). In recent years, some important transcription factor families including AP2/ERFs, WRKYs, MYBs, and NACs families were determined to regulate the expression of biologically related genes in plants (Meraj et al., 2020). In the current research, we found that TFs belonging to bZIPs, WRKYs, MYBs, and AP2/ERFs are specifically expressed and have higher basal expression (0h) in resistant grape cultivars (Figure 9 and Supplementary Table 6). Interestingly, the expression of the bZIP factor increased during *Ustilago maydis* infection and exhibited a prominent function in response to pathogen attack in maize (Wei et al., 2012). The CaWRKY27 from pepper also improved resistance against necrotrophic pathogen *Ralstonia solanacearum* (Dang et al., 2014). Overexpression of MtERF1-1 in roots of the legume *Medicago truncatula* (barrel medic) increased resistance to *Rhizoctonia solani* and against the oomycete *Phytophthora medicaginis* (Anderson et al., 2010). These results indicate that transcription factors may interact with jasmonic acid and salicylic acid to regulate the process of grape resistance to downy mildew.

## Conclusion

Here, based on our transcriptome database (Supplementary Figure 4), we constructed a hypothetical model of two grape cultivars (*Viti’s vinifera* cv. “Zitian Seedless” and Kober “5BB”) and showed the similarities and differences during defense process against *P. viticola* (Figure 9). In the early stage of infection, we observed *P. viticola* zoospores in stomata of both resistant/susceptible grape leaf tissues. The different response pathways, including ROS, sphingolipids, flavonoids, cytoskeleton, calcium channels, and MAPK cascade pathways were activated under pathogen infection, although the levels of their activation that could alter according to the types of cultivars is disease-resistant. In “Kober 5BB” grapes, pathogen receptors (RLKs, NBS-LRRs, etc.) specifically recognize pathogenic secretions (effectors) in the host cell. Activated pathogen receptors (including R genes) trigger stronger defense signaling pathways based on PTI, including MAPK and Ca^2+^ signaling pathways. Meanwhile, the production of ROS (hydrogen peroxide) is also regulated more by these two signaling pathways. The activated R genes further regulate and activate proteases related to cell death execution, triggering the cascade effect of HR, which would be an efficient strategy against biotrophic pathogen infection. According to the final morphological characteristics, we could observe resistance against downy mildew. In the aspect of susceptible “Zitian seedless” grape, due to the lack of defense mechanisms at the ETI level, the cells are quickly eroded/utilized by pathogens. The specific manifestation is that the cell wall tissue, sugar, and photosynthetic related processes are blocked, and the expression of related genes is downregulated.

## Data Availability Statement

The datasets presented in this study can be found in online repositories. The names of the repository/repositories and accession number(s) can be found in the article/Supplementary Material.

## Author Contributions

PG and JF: conceived and designed the experiments. JK, PG, RB, MG, YZ, and LS: analyzed the data. JK, PG, and ES: conducted the experiments and wrote the manuscript. ES, PG, LS, and JF: revised the manuscript. All authors worked on developing the final manuscript and read and approved it.

## Conflict of Interest

The authors declare that the research was conducted in the absence of any commercial or financial relationships that could be construed as a potential conflict of interest.

## Publisher’s Note

All claims expressed in this article are solely those of the authors and do not necessarily represent those of their affiliated organizations, or those of the publisher, the editors and the reviewers. Any product that may be evaluated in this article, or claim that may be made by its manufacturer, is not guaranteed or endorsed by the publisher.

## Footnotes

1
http://www.novogene.cn/

2
ftp://ftp.ensemblgenomes.org/pub/release-23/plants/gtf/vitis_vinifera/

3
http://ccb.jhu.edu/software/hisat2/index.shtml

4
http://bioinformatics.psb.ugent.be/webtools/Venn/

5
http://mapman.gabipd.org/web/guest/mapmanstore

6
https://string-db.org/
