# Supplementary material for: Transcriptional Profiling of Resistant and Susceptible Cultivars of Grapevine (Vitis L.) Reveals Hypersensitive Responses to Plasmopara viticola
Source: Front Microbiol. 2022 Apr 25;13:846504. doi: 10.3389/fmicb.2022.846504 (PMC9097084; doi:10.3389/fmicb.2022.846504)
Supplement: Supplementary file 2 [file Data_Sheet_2.pdf]

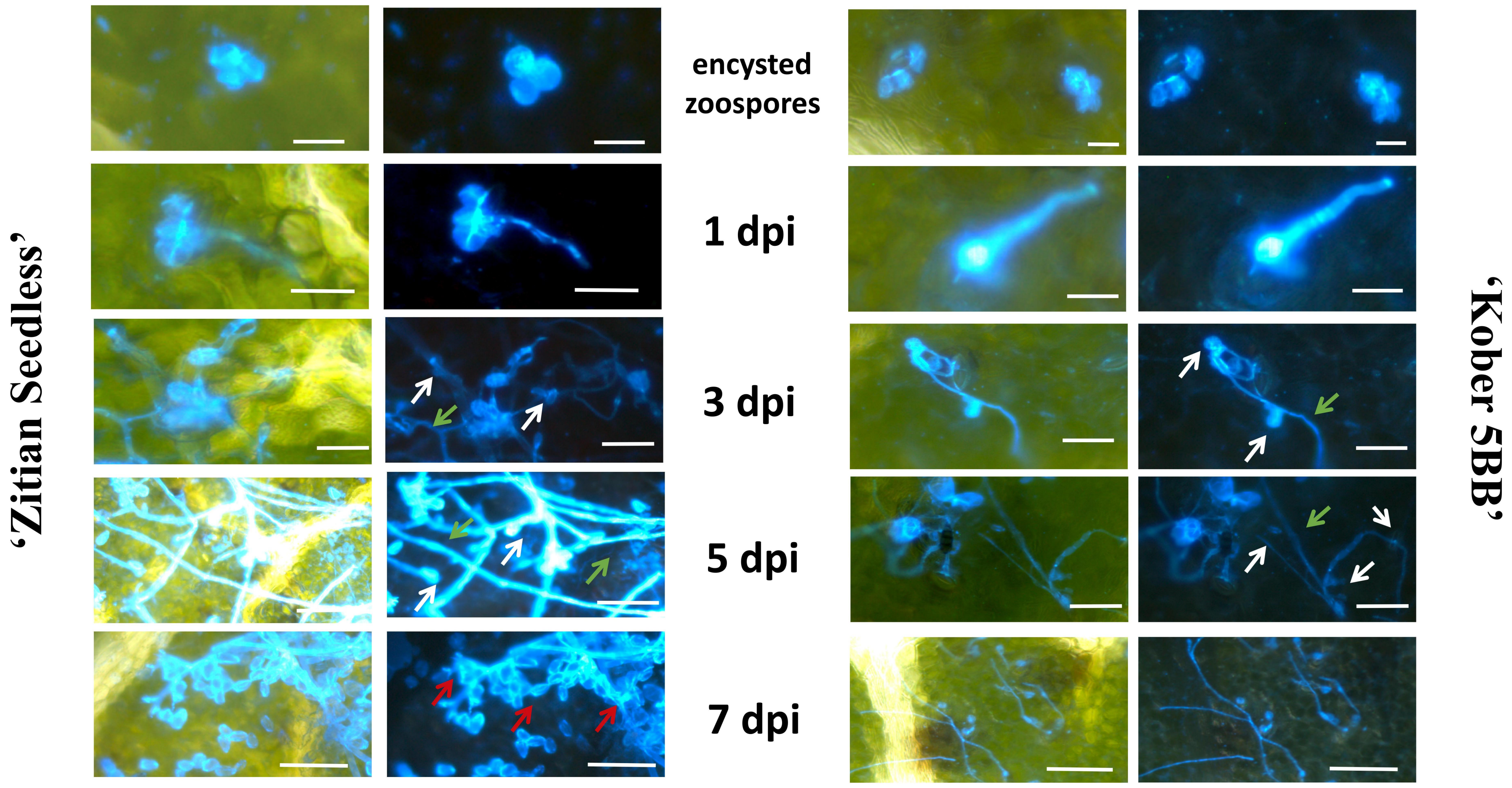

Supplementary Figure S1 Morphological structure of *P.viticola* in the two grape varieties 'Zitian Seedless' and 'Kober 5BB' infected by downy mildew strains BS-4-MW. Encysted zoospores: within 2 hours of infestation, zoospores were observed to invade the stomata; dpi: day post infestation; green arrows:invasive hyphae; white arrows: haustoria; green arrow: red arrows: sporangium; Bars: 20 µm (first two lines)，40µm (the 3rd and 4th lines), 100µm (last line).

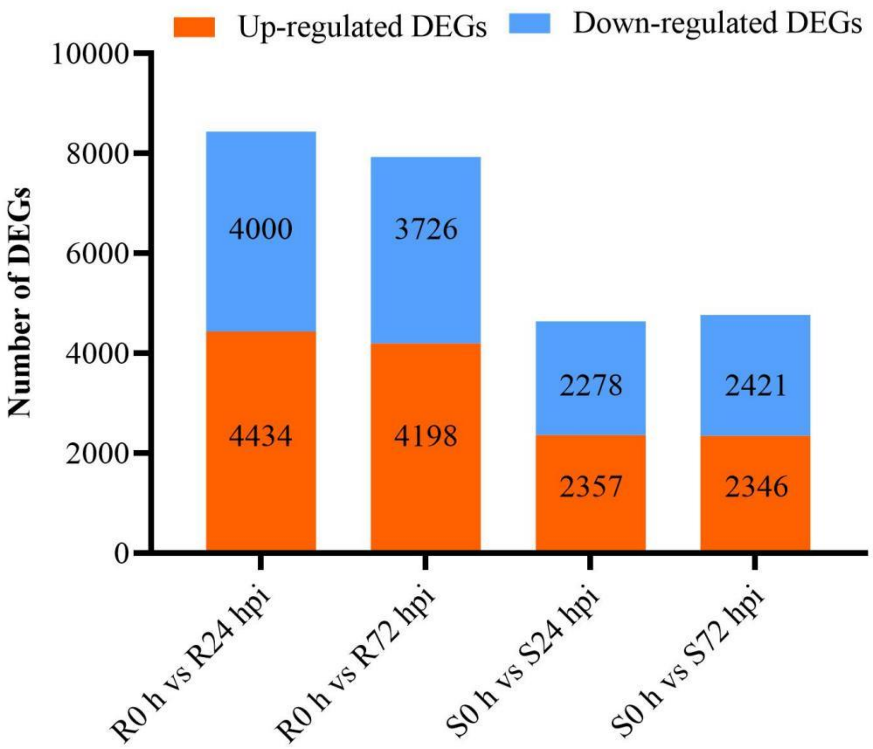

Supplementary Figure S2 Number of up- and down-regulated genes in 'Kober 5BB' and 'Zitian Seedless' following inoculation with *P.viticola* BS-4-MW.

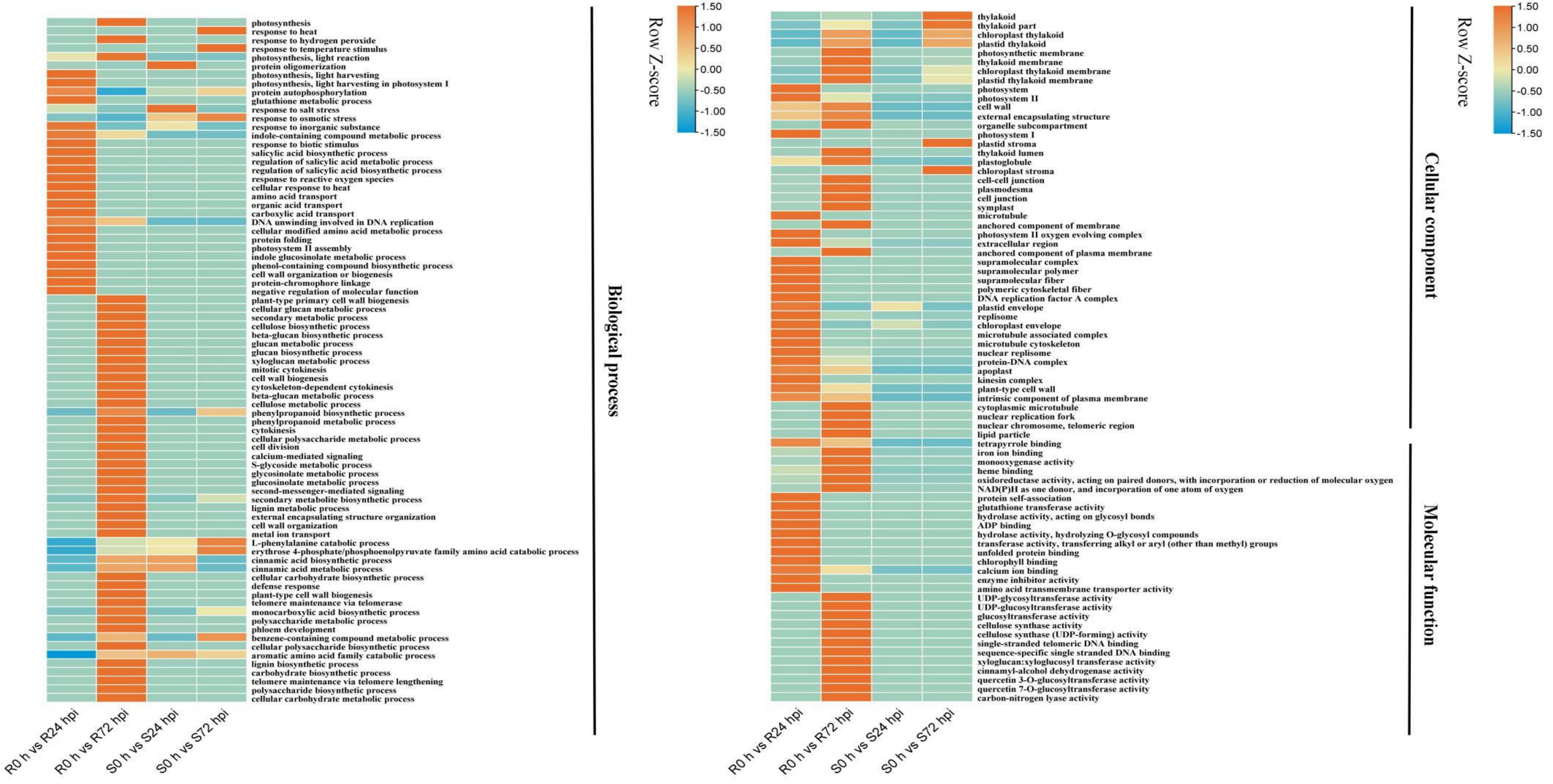

Supplementary Figure S3 Heatmap of enriched GO terms identified from DEGs in resistant ('Kober 5BB') and susceptible ('Zitian Seedless') grape cultivars. Terms are considered enriched when $P < 0.05$. R resistant cultivar *Vitis* rootstocks 'Kober 5BB'. S *Vitis vinifera cv.* 'Zitian Seedless'.

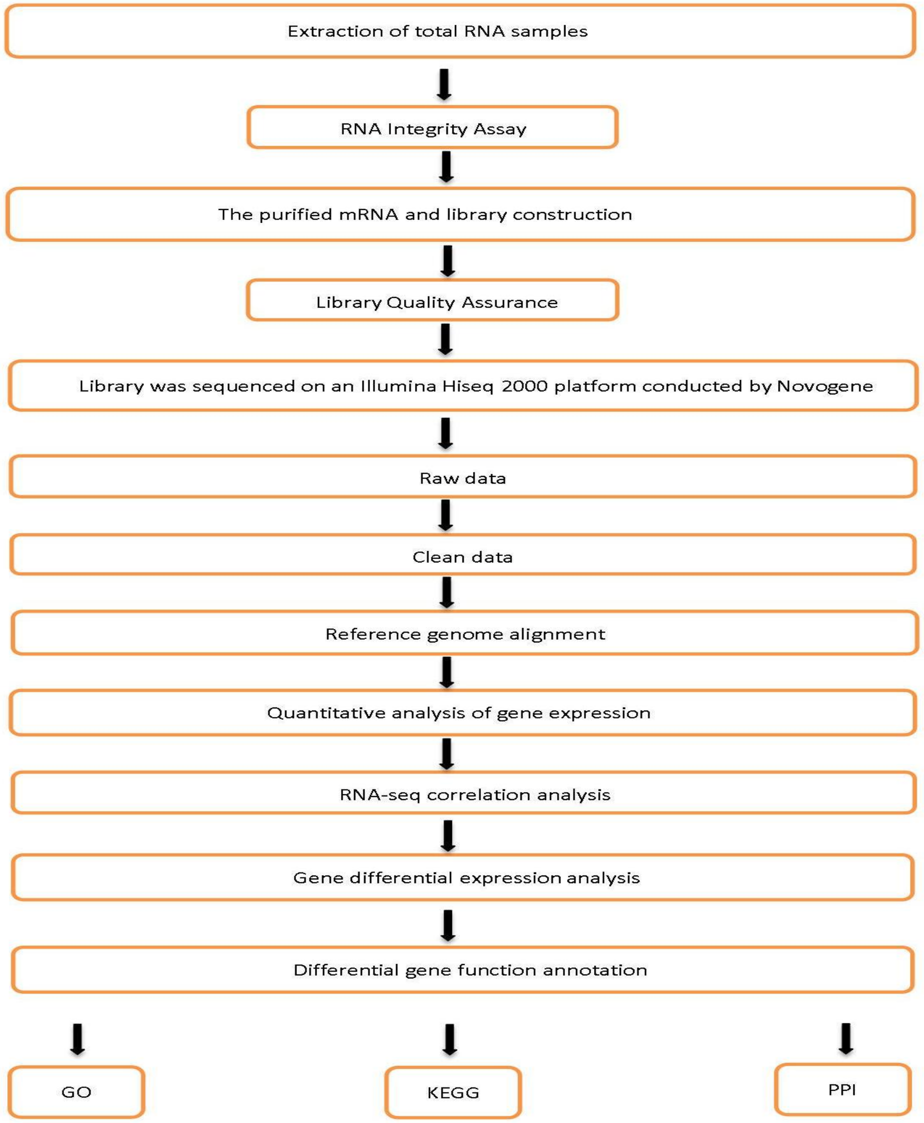

Supplementary Figure S4 A flowchart to summarize the transcriptome data and analysis approach.
